# Beta-Cell Specific Deletion of *Dicer1* Leads to Defective Insulin Secretion and Diabetes Mellitus

**DOI:** 10.1371/journal.pone.0029166

**Published:** 2011-12-27

**Authors:** Martins Kalis, Caroline Bolmeson, Jonathan L. S. Esguerra, Shashank Gupta, Anna Edlund, Neivis Tormo-Badia, Dina Speidel, Dan Holmberg, Sofia Mayans, Nelson K. S. Khoo, Anna Wendt, Lena Eliasson, Corrado M. Cilio

**Affiliations:** 1 Cellular Autoimmunity Unit, Lund University Diabetes Center, Department of Clinical Sciences, Lund University, Malmö University Hospital, Malmö, Sweden; 2 Islet Cell Exocytosis, Lund University Diabetes Center, Department of Clinical Sciences, Lund University, Malmö University Hospital, Malmö, Sweden; 3 Department of Medical Genetics, Umeå University, Umeå, Sweden; 4 Department of Disease Biology, Faculty of Life Science, Copenhagen University, Copenhagen, Denmark; University of Bremen, Germany

## Abstract

Mature microRNAs (miRNAs), derived through cleavage of pre-miRNAs by the Dicer1 enzyme, regulate protein expression in many cell-types including cells in the pancreatic islets of Langerhans. To investigate the importance of miRNAs in mouse insulin secreting β-cells, we have generated mice with a β-cells specific disruption of the *Dicer1* gene using the Cre-lox system controlled by the rat insulin promoter (RIP). In contrast to their normoglycaemic control littermates (RIP-Cre*^+/−^ Dicer1* ^Δ/wt^), RIP-Cre^+/−^ *Dicer1^flox/flox^* mice (RIP-Cre *Dicer1* ^Δ/Δ^) developed progressive hyperglycaemia and full-blown diabetes mellitus in adulthood that recapitulated the natural history of the spontaneous disease in mice. Reduced *insulin* gene expression and concomitant reduced insulin secretion preceded the hyperglycaemic state and diabetes development. Immunohistochemical, flow cytometric and ultrastructural analyses revealed altered islet morphology, marked decreased β-cell mass, reduced numbers of granules within the β-cells and reduced granule docking in adult RIP-Cre *Dicer1* ^Δ/Δ^ mice. β-cell specific *Dicer1* deletion did not appear to disrupt fetal and neonatal β-cell development as 2-week old RIP-Cre *Dicer1* ^Δ/Δ^ mice showed ultrastructurally normal β-cells and intact insulin secretion. In conclusion, we have demonstrated that a β-cell specific disruption of the miRNAs network, although allowing for apparently normal β-cell development, leads to progressive impairment of insulin secretion, glucose homeostasis and diabetes development.

## Introduction

The insulin-secreting function of pancreatic β-cells is one of the most critical aspects in the development of diabetes [1] and insulin release from pancreatic β-cells plays an essential role in blood glucose homeostasis [2]. Insulin is synthesized through transcription and translation of the insulin gene into preproinsulin and cleavage of the signal peptide and the C-peptide during the maturation process [3]. Both transcription and translation of insulin are glucose-dependent processes. Insulin gene expression is increased through glucose-dependent activation of specific transcription factors [4] and posttranscriptional regulation of insulin involves glucose-dependent insulin mRNA stabilization and translation [5]. Mature insulin can remain within the insulin granules for several days [6] before it is transported out to the plasma membrane. A large proportion of the granules reside in a reserve pool within the β-cell whereas <1% of the granules are docked and primed at the plasma membrane [7]. We and others have hypothesized that the release of primed granules correlates with the 1^st^ phase insulin secretion while release of granules within the larger reserve pool constitutes the release during the 2^nd^ phase insulin secretion [8], [9].

MicroRNAs are a class of 19–25 nucleotide (nt)-long noncoding RNA molecules that are involved in regulation of gene expression [10]. Mature miRNAs are processed from double-stranded hairpin precursors by Drosha and Dicer enzymes [11]. Only one Dicer enzyme is present in mammals and is responsible for the final step in miRNA processing [12]. Disruption of the catalytic process mediated by Dicer results in lack of mature miRNAs, leading to altered target gene expression [11]. Germline deletion of *Dicer1* is embryonically lethal in mice [13], [14] and results in altered embryonic stem cell differentiation [15].

Several miRNAs regulating different parts of the insulin-secreting process have been identified. The β-cell enriched miRNA miR-375 has been demonstrated to influence insulin mRNA levels [16] as well as the exocytotic process [17]. Moreover, the level of miRNA-375, together with miRNA-127-3p and miR-184 is positively correlated to insulin mRNA levels in islets from human donors and the association between these miRNAs and β-cell function was deranged in islets from glucose intolerant donors [18]. MiR-375 has also been implicated in β-cell expansion and proliferation [19]. Recently, several other miRNAs including miR-24 and miR-182 was implicated to be important for insulin biosynthesis [20]. Other miRNAs directly regulating insulin secretion affecting the expression of proteins involved in the exocytotic process include miR-124a and miR-96 [21]. Indeed, it was recently shown that the predicted targets of a number of miRNAs overexpressed in the pancreatic islets of the type 2 diabetes model, Goto-Kakizaki rat, were shown to be enriched for genes with central roles in exocytosis, including Syntaxin-binding protein 1 (Stxbp1) shown experimentally to be a target of rno-miR-335 [22]. In addition, miRNAs have been suggested to contribute to fatty acid-induced β-cell dysfunction [23].

β-cell specific *Dicer1* deletion controlled by the Pdx1 promoter results in aberrant pancreas development (specifically in the insulin-secreting β-cells) and neonatal death [24]. Inactivation of *Dicer1* in adult life leads to development of diabetes due to reduced insulin expression [20]. However, the effect of progressive loss of Dicer 1 during life has not yet been determined. We have studied the role of miRNAs in β-cell function and in the development of diabetes *in vivo* by specifically deleting *Dicer1* in pancreatic β-cells under the RIP-promoter. Our results show that disruption of miRNA processing in mouse pancreatic β-cells, while compatible with β-cell development, leads to defective insulin-secreting function and progressive overt diabetes mellitus.

## Results

### β-cell specific deletion of *Dicer1* gene results in diabetes development

**Figure 1:**
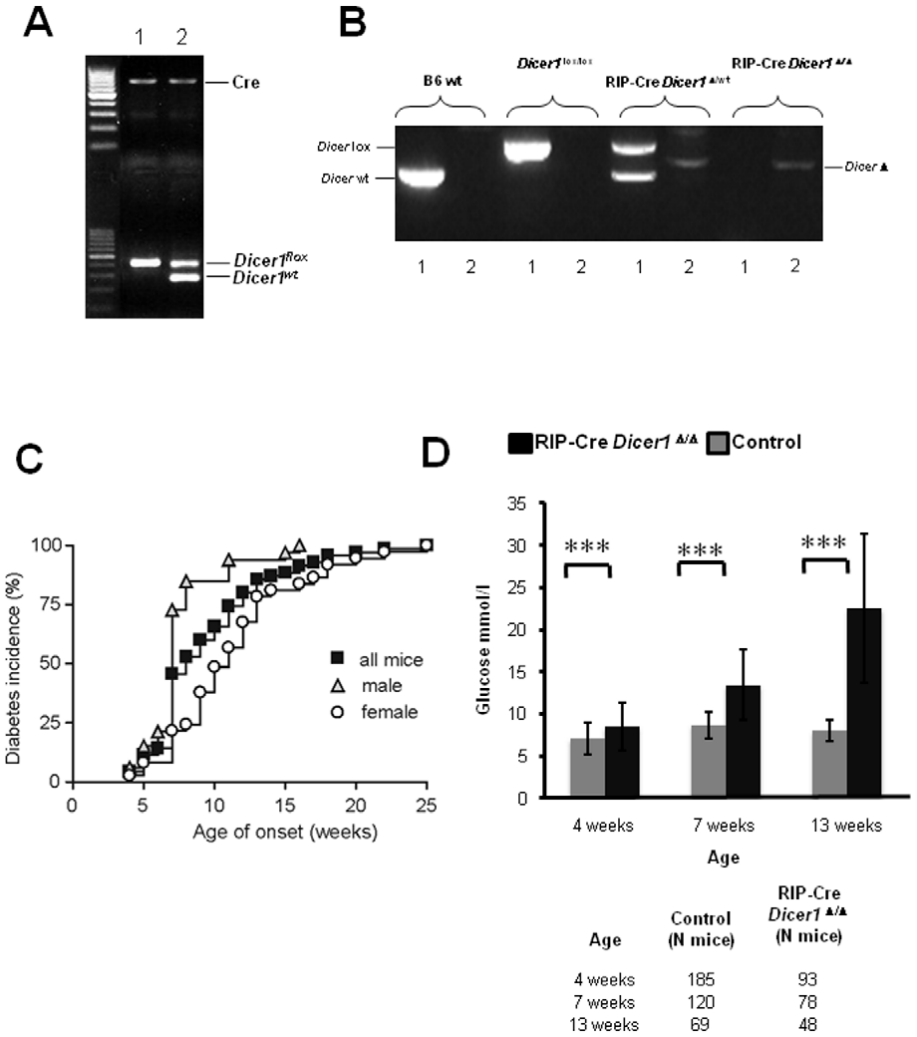
RIP-Cre Dicer1*^Δ/Δ^* mice develop diabetes during the first 25 weeks of age. A. PCR analysis of tail genomic DNA showing the genotypes of the mice used in the experiments, lane 1 RIP-Cre *Dicer1^Δ/Δ^* and lane 2 their control littermates RIP-Cre *Dicer1^Δ^* ^/wt^ B. PCR analysis of genomic DNA from sorted β-cells demonstrating deletion of both alleles of *Dicer1* gene in RIP-Cre *Dicer1^Δ/Δ^* and deletion of only one allele in control littermates RIP-Cre *Dicer1^Δ^* ^/wt^ mice. Numbers below lanes represent the two PCR reactions (one for each allele) for each mouse strain. C. Cumulative diabetes incidence in RIP-Cre *Dicer1^Δ/Δ^* mice. Glucose was measured every week from the age of 3 weeks using a glucometer. Control littermates never develop diabetes or hyperglycaemia and are therefore not shown in the figure. D. Plasma blood glucose levels (mmol/l) measured in 4, 7 and 13 weeks old RIP-Cre *Dicer1^Δ/Δ^* mice and littermate controls as indicated. The number of animals within the different groups is indicated below. Data are presented as mean±sd. ***P<0.001.

To investigate the effect of *Dicer1* deficiency on diabetes development, we generated mice with a β-cell specific *Dicer1* deletion from mid gestation (E9–11.5) using the Cre-lox system (RIP-Cre *Dicer1^Δ/Δ^*) by crossing RIP-Cre transgenic with Dicer^flox/flox^ mice (Fig. 1A and B). The possibility of RIP-Cre mediated *Dicer1* deletion in brain [25] and the possible effects in feeding behaviour and body weight was excluded by measuring *Dicer1* expression and daily measurement of body weight (Fig. S1A–B). Feeding behavior, measured by controlling food consumption, also remained unchanged between the two mouse strains (Fig. S1C). The incidence of spontaneous diabetes was determined in RIP-Cre *Dicer1^Δ/Δ^* mice and control littermates (RIP-Cre *Dicer1* ^wt/Δ^) from 2 weeks of age. Mice were considered diabetic when blood glucose levels were ≥13.5 mmol/l on two consecutive measurements. The onset of diabetes was dated to the first of these two measurements. As shown in Fig. 1C, by 12 weeks of age, 80% of *Dicer1* deficient mice (n = 70) developed diabetes and, by 25 weeks of age, 100% of mice were diabetic. Male mice (n = 33) developed diabetes faster compared to females (n = 37; p = 0.0005). Fig. 1D shows the measured plasma glucose levels in 4, 7 and 13 weeks old mice. Control littermate mice (n = 70) always remained normoglycaemic.

### In vivo impairment of glucose homeostasis in RIP-Cre *Dicer1 ^Δ/Δ^* mice

**Figure 2:**
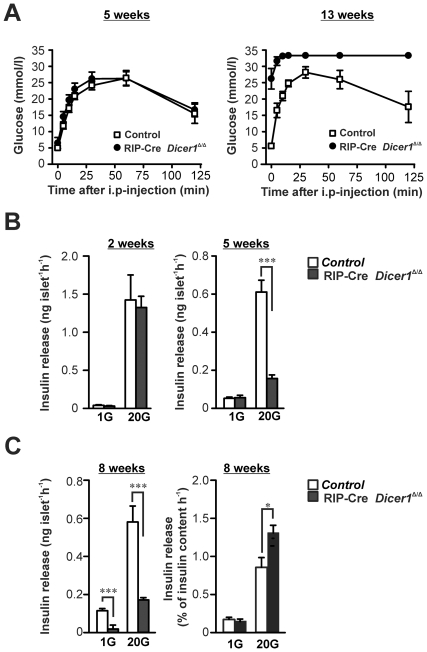
Impaired in vivo glucose tolerance and in vitro insulin secretion in RIP-Cre Dicer1*^Δ/Δ^* mice. A. *In vivo* glucose tolerance test was performed on 6 control littermates and 5 RIP-Cre *Dicer1^Δ/Δ^* females at 4–5 weeks of age (left) and 6 control littermates and 6 RIP-Cre *Dicer1^Δ/Δ^* females at 13 weeks of age (right). Note that the glucose levels of the 13 weeks old RIP-Cre *Dicer1^Δ/Δ^* mice reached levels not measurable by the glucometer. Data are mean±SEM. B. Insulin secretion from isolated pancreatic islets was measured after 1 h incubation in 1 mM glucose (1 G) or 20 mM glucose (20 G) in 2 and 5–6 weeks old littermate control (white bar) or RIP-Cre *Dicer1^Δ/Δ^* mice (black bar). C. (*Left*) As in B, but insulin secretion was measured in 7–8 weeks old mice. (*Right*) Insulin release normalized to insulin content. Data are mean±SEM of 15–21 experiments in 3–5 animals. *P<0.05; ***P<0.001.

To investigate the efficiency of RIP-Cre *Dicer1^Δ/Δ^* β-cells to maintain *in vivo* glucose homeostasis after glucose challenge, we performed the intraperitoneal glucose tolerance test (ipGTT). No differences in glucose tolerance between RIP-Cre *Dicer1^Δ/Δ^* and control mice (Fig. 2A) were observed in 4–5 weeks old mice. By contrast, at 13 weeks of age, RIP-Cre *Dicer1^Δ/Δ^* mice showed extremely impaired glucose tolerance compared to control mice (P<0.0001, Fig. 2A).

### Impaired insulin secretion precedes hyperglycaemia in RIP-Cre *Dicer1^Δ/Δ^* mice

To investigate the effects of the β-cell-specific *Dicer1* deletion on insulin secretion *in vitro*, we performed batch-incubations of pancreatic islets. Islets were incubated at basal (1 mM) or stimulating (20 mM) glucose concentration for 1 hour. Insulin secretion in response to basal glucose concentration was normal in young (2 and 5 weeks old) RIP-Cre *Dicer1^Δ/Δ^* mice, but significantly reduced in 8 weeks old RIP-Cre *Dicer1^Δ/Δ^* mice (P<0.0001, Fig. 2C). Insulin secretion in response to 20 mM glucose was normal in 2 weeks old RIP-Cre *Dicer1^Δ/Δ^* mice, but reduced by over 70% in 5 and 8 weeks old RIP-Cre *Dicer1^Δ/Δ^* mice (P<0.0001, Fig. 2B and C). However, upon normalization for insulin content insulin secretion levels in 8 weeks old mice actually increased compared to those of controls (P<0.05, Fig. 2C). These results indicate a defect in either insulin production, β-cell survival or both in the RIP-Cre *Dicer1^Δ/Δ^* mice.

### *Dicer1* disruption in β-cells leads to morphological changes in pancreatic islets

**Figure 3:**
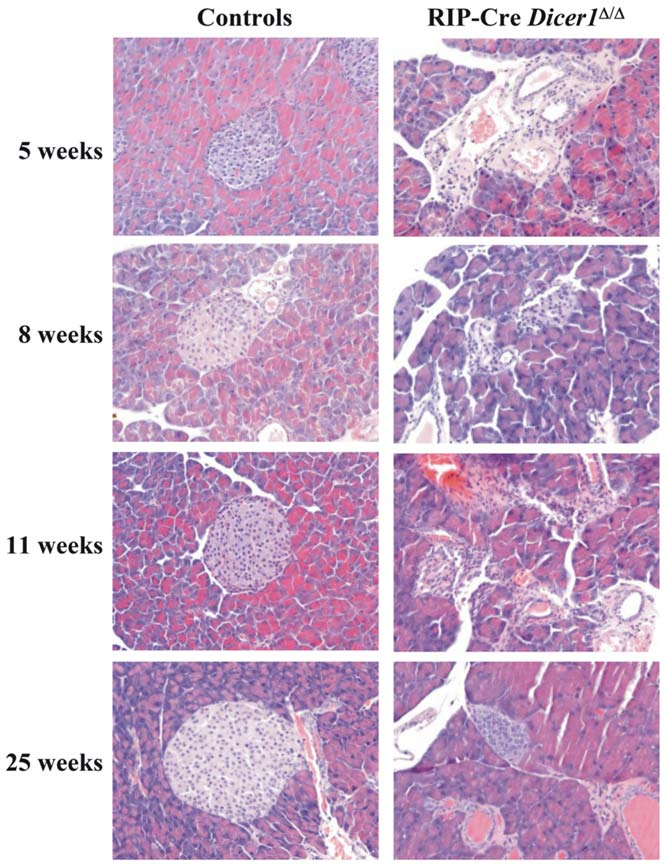
Altered islet morphology in RIP-Cre Dicer1*^Δ/Δ^* mice. H-E stainings of 5, 8, 11 and 25 weeks old littermate controls (*left*) or RIP-Cre *Dicer1^Δ/Δ^* (*right*) mice. Representative islets of more than 5 mice per group and age are shown. All images are 20× magnification.

The histological phenotype of the pancreatic islets was examined using H-E staining. The analysis performed on RIP-Cre *Dicer1^Δ/Δ^* and control pancreas at 5, 8, 11 and 25 weeks of age (Fig. 3) showed that islet morphology was clearly altered already at 5 weeks of age in RIP-Cre *Dicer1^Δ/Δ^* mice. In 25 weeks old RIP-Cre *Dicer1^Δ/Δ^* mice the number of islets was dramatically decreased and in general smaller compared to littermate controls.

### *Dicer1* disruption in β-cells leads to progressive decrease of insulin producing cells and total β-cell mass

**Figure 4:**
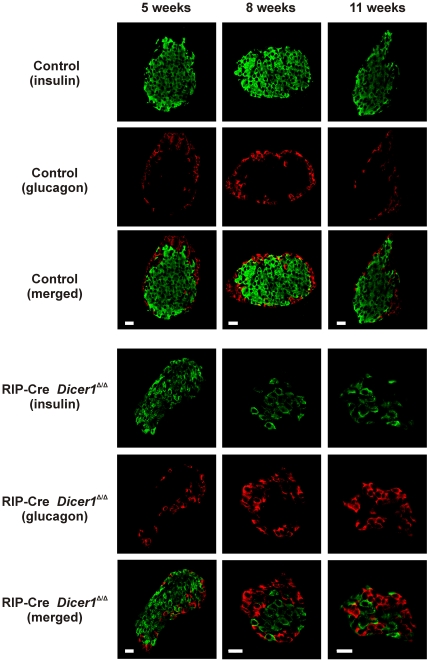
Altered cellular morphology in RIP-Cre Dicer1*^Δ/Δ^* islets with increasing age. Confocal microscopy analysis of insulin and glucagon staining was performed on islets from 5, 8 and 11 weeks old control littermates (*upper panels*) and RIP-Cre *Dicer1^Δ/Δ^* (*lower panels*) mice. Insulin and glucagon staining can be observed as indicated. Notice the deformation of the RIP-Cre *Dicer1^Δ/Δ^* islet and the increased number of α-cells relative to the reduced number of β-cells. Scale bar 20 µm.

To visualize insulin-producing cells *in situ*, we performed immunohistochemical analysis using confocal microscopy (Fig. 4). At 5 weeks of age, the insulin to glucagon ratio (R) was not significantly different between RIP-Cre *Dicer1^Δ/Δ^* (R = 3.3±0.7, n = 6) and control littermate mice (R = 4.9±1.1, n = 5). In 8 to 11 weeks old mice we observed a clear reduction of β-cells in RIP-Cre *Dicer1^Δ/Δ^* mice compared to control littermates with a relative increase in the number of α-cells, and the ratio was 0.8±0.2 (n = 12) in the RIP-Cre *Dicer1^Δ/Δ^* mice islets and 3.6±0.6 in control littermates (n = 12; p<0.001 control littermates *vs* RIP-Cre *Dicer1^Δ/Δ^*).

**Figure 5:**
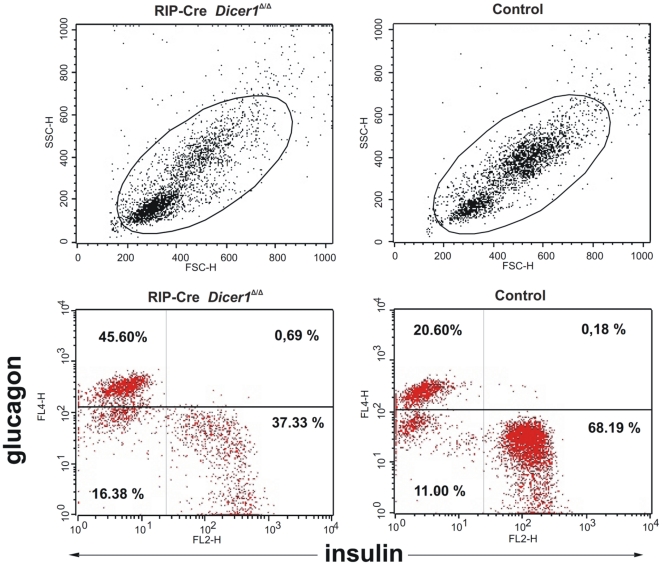
Decreased insulin-producing cells in RIP-Cre Dicer1*^Δ/Δ^* islets. Islet cells suspension from 50 islets from 8 weeks old littermates and RIP-Cre *Dicer1^Δ/Δ^* mice were stained for insulin and glucagon and analyzed by flow cytometry. Side scatter (SSC) and forward scatter (FSC) analysis of islet cells and the respective gate used to identify live cells. Littermate control islet cells contained significantly more insulin-positive cells (P<0.001) and less glucagon-positive cells (P<0.05) than the RIP-Cre *Dicer1^Δ/Δ^* islet cells. Representative of 3 independent experiments.

Both the morphological changes and the observed decrease in the β-cell to α-cell ratio in RIP-Cre *Dicer1^Δ/Δ^* islets could be related to a reduced number of β-cells. To more accurately quantify the frequency of pancreatic β- and α-cells, we performed flow cytometric analysis of dispersed islets on 8 to 11 weeks old RIP-Cre *Dicer1^Δ/Δ^* mice and age-matched control littermates using antibodies specific for insulin and glucagon. As shown in Fig. 5, we confirmed a significant reduction of β-cells (from 68±1% insulin positive cells in control littermates to 42±3% in RIP-Cre *Dicer1^Δ/Δ^* mice; P<0.001, n = 3) with a relative increase of α-cells (from 20±2% glucagon positive cells in control littermates to 38±5% in RIP-Cre *Dicer1^Δ/Δ^* mice; P<0.05, n = 3).

**Figure 6:**
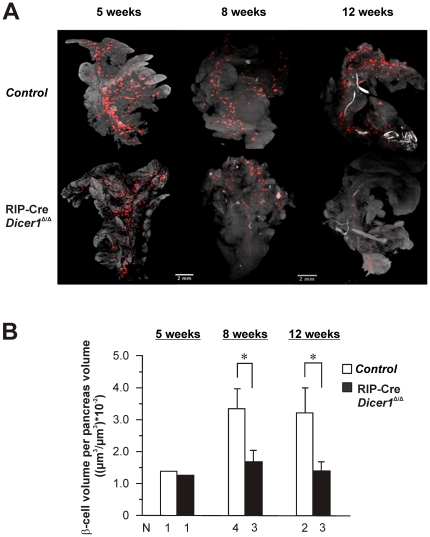
Decreased total β-cell mass in RIP-Cre Dicer1*^Δ/Δ^* visualised by optical projection tomography. A. Optical tomographic sections based on insulin-labeling of adult pancreas of 5, 8 and 12 weeks old RIP-Cre *Dicer1* and control littermate mice. A clear decrease of total β-cells can be observed in pancreas from 8 and 12 weeks old hyperglycemic in RIP-Cre *Dicer1^Δ/Δ^* mice. Notice that the image shown is a 2D image and that the OPT technique generate a 3D reconstruction of the whole pancreas. B. Quantification of β-cell volume per pancreas volume from 5, 8 and 12 week old RIP-Cre *Dicer1* and control littermate mice. The number of animals used in the analysis of the different groups is given below the columns. Data are presented as mean±SEM for the 8 and 12 week old mice. *P<0.05.

Based on these results, we next performed Optical Projection Tomography (OPT) [26] to study the total β-cell mass in whole-mounted pancreas 3-dimensionally. As shown in Fig. 6, there was no significant difference in total number of insulin-producing cells between RIP-Cre *Dicer1^Δ/Δ^* and control littermates at 5 weeks of age. However, we could undoubtedly demonstrate a significant reduction of total insulin-producing cells in RIP-Cre *Dicer1^Δ/Δ^* compared to littermate control mice in 8 and 12 week old mice. Quantification of the number of islets per pancreas in 8–12 week old mice revealed a significant decrease from 2300±451 islets/pancreas (N = 6 mice) in control mice to 1485±378 islets/pancreas (N = 6 mice; p = 0.0035 *vs* control mice) in RIP-Cre *Dicer1^Δ/Δ^*. Likewise, β-cell mass measured as β-cell volume per whole pancreas volume [26], [27] was reduced in 8 and 12 week old RIP-Cre *Dicer1^Δ/Δ^* mice by ≥50% compared with their control littermates of the same age (Fig. 6B).

### Ultrastructural analysis reveals β-cell loss and reduction of β-cell secretory granules

**Figure 7:**
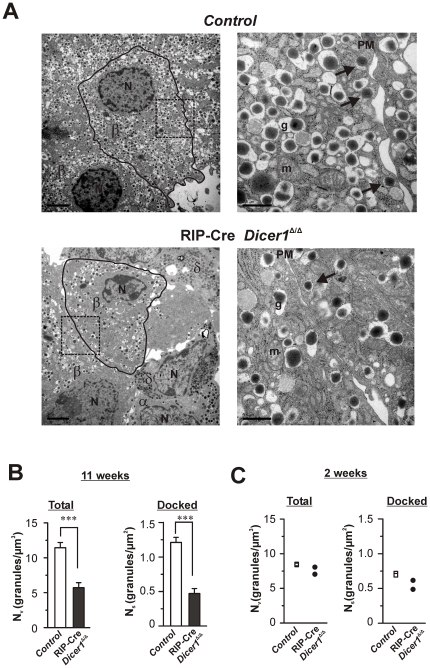
Ultrastructural changes in RIP-Cre Dicer1*^Δ/Δ^* β-cells. A. Electron micrograph of β-cells from 11 weeks old control (*top*) and RIP-Cre *Dicer1^Δ/Δ^* (*bottom*) mice. In the *left* images (low magnification) the plasma membrane surrounding the analyzed β-cell is marked by a black line. The area within the dotted rectangle is shown in the higher magnification images to the *right*. Docked granules are marked with black arrows in the images to the *right*. Scale bars 2 µm (*left images*) and 0.5 µm (*right images*). β: β-cell; α: α-cell; δ: δ-cell; N: nucleus; g: granule; m:mitochondria. B. Histograms of the total amount of granules (*left*) and the number of docked granules (*right*) within β-cells measured as volume density N_V_ (granules/µm^3^) and surface density N_s_ (granules/µm^2^), respectively. Analysis was performed on β-cells within islets from 11 weeks old control littermates (white bar) and RIP-Cre *Dicer1^Δ/Δ^* mice (black bar). Data is expressed as mean±SEM of 23 cells in each group. ***P<0.001. The embedded islets where from N = 4 mice. C. As in B, but analysis was performed on β-cells within islets from 2 week old control littermates (white bar) and RIP-Cre *Dicer1^Δ/Δ^* mice (black bar). Data are from N = 2 mice each for control and RIP-Cre *Dicer1^Δ/Δ^* mice, and the mean from each of the individual mice are presented in the graph.

To better characterize the consequences of *Dicer1* disruption at single cell level, we performed transmission electron microscopy (TEM). In 11 weeks old RIP-Cre *Dicer1^Δ/Δ^* mice, the electron micrographs revealed an obvious reduction of the number of β-cells within the islets compared to the control islets. The electron micrographs indicated that the few remaining β-cells in RIP-Cre *Dicer1^Δ/Δ^* had less insulin granules than their control littermates (Fig. 7A). This was confirmed by ultrastructural analysis (Fig. 7B) which revealed ∼50% less granules in the RIP-Cre *Dicer1^Δ/Δ^* compared to the controls when measured as volume-density (N_v_; Fig. 7B, *left*). This reduction was specific for the β-cells and could not be observed in non-β-cells (data not shown). The number of docked granules expressed as the surface density (N_s_) was also severely reduced in β-cells from 11 week old RIP-Cre *Dicer1^Δ/Δ^* mice compared to control littermates (Fig. 7B, *right*). At 2 weeks of age this phenotypic feature had not appeared (Fig S2) and the β-cells in RIP-Cre *Dicer1^Δ/Δ^* mice had a similar granular volume density N_v_ and surface density N_s_ as control littermates (Fig. 7C).

### *Dicer1* knockdown dramatically reduce *insulin* gene expression in isolated β-cells

**Figure 8:**
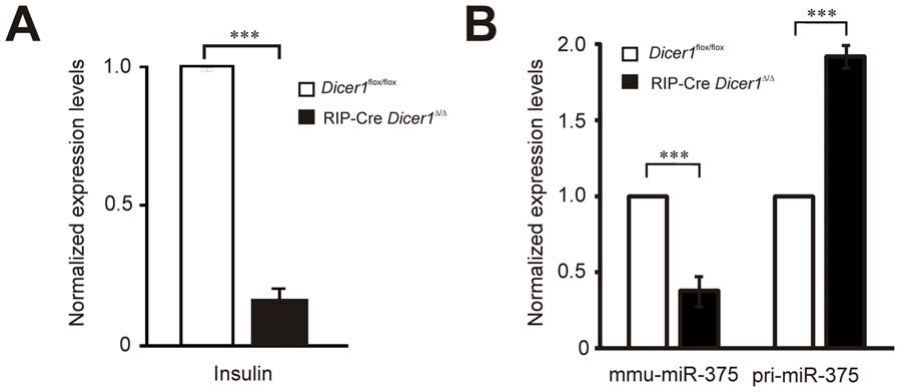
Insulin, miR-375 and pri-miR-375 expression in sorted β-cells. Pancreatic islets were isolated from 7 RIP-Cre *Dicer1^Δ/Δ^* and 7 littermate control mice. β-cells were sorted using flow cytometry to >98% purity. Total RNA was extracted and used for qPCR and array hybridization. A. Insulin mRNA expression level is reduced in RIP-Cre *Dicer1^Δ/Δ^*, B. Mature miR-375 is significantly reduced while the pri-miR-375 species accumulates in the knockout. ***P<0.001.

We next tested whether the level of *Dicer1* deletion affect *insulin* expression in sorted β-cells. Purified islets from 6 to 8 weeks old non-diabetic RIP-Cre *Dicer1^Δ/Δ^* (n = 7) and control littermates (n = 7) were pooled and dissociated before being stained with Newport Green-Ac and sorted by flow cytometry as described in material and methods. The purity after sorting was >95% as determined by insulin staining and immunofluorescence microscopy (data not shown). In line with the data showed above, we could demonstrate by qPCR a 90% decrease of *insulin* gene expression in RIP-Cre *Dicer1^Δ/Δ^* compared to littermate control mice (Fig. 8 A). To verify that the knock-down of dicer effects miRNA expression we measured the expression of the β-cell specific miRNA, mir-375, and found that it was reduced by 70% in the RIP-Cre *Dicer1^Δ/Δ^* compared to littermate control mice (Fig. 8 B). This was accompanied by an accumulation of pri-miRNA-375 in the RIP-Cre *Dicer1^Δ/Δ^* β-cells (Fig. 8B).

To investigate the effect of *Dicer1* knockdown on the global miRNA level, we hybridized total RNA from sorted β-cells of wildtype, *Dicer1* ^flox/flox^ and RIP-Cre *Dicer1^Δ/Δ^* into locked nucleic acid (LNA)-based arrays. Owing to the limited starting material, we could only perform the experiments once for each sample and we generally observed low signal intensities from the arrays. Of the 60 quantifiable miRNA signals, 26 miRNAs were not detectable in RIP-Cre *Dicer1^Δ/Δ^* (Fig. S3). These include the islet-specific miR-375 and another abundant islet miRNA, miR-7a [28]. Surprisingly, 14 miRNAs were similarly expressed in the knockout animals as in the controls while 20 miRNAs were found to be exclusively expressed in the former (Fig. S3). Our array data indicate the existence of both Dicer1-dependent and -*in*dependent maturation pathway of miRNAs in line with previous studies [29], [30]. However, our array data cannot conclusively support the extent of a Dicer1-*in*dependent pathway without further validation.

### No evidence for altered β-cell proliferation or apoptosis in RIP-Cre *Dicer1^Δ/Δ^* mice

**Figure 9:**
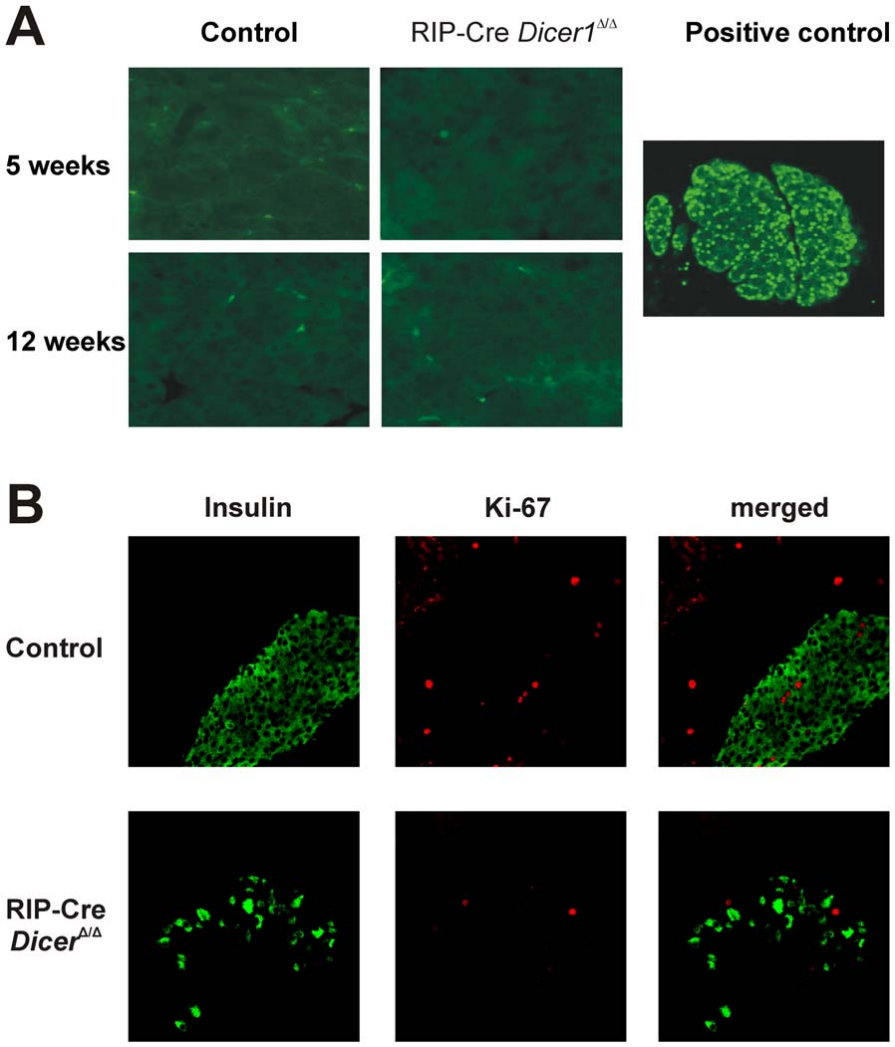
No evidence of increased β-cell apoptosis and altered proliferation in RIP-Cre *Dicer1^Δ/Δ^* mice. A. β-cell apoptosis was estimated using *in situ* TUNEL assay from 8–12 weeks old RIP-Cre *Dicer1^Δ/Δ^* and littermate control mice (right). No significant apoptosis could be detected in any islets observed. Nuclease-treated pancreas section from age matched littermate control mice was used as positive control for TUNEL staining and is depicted on the left. These are representative of several sections of individual pancreases. B. β-cell proliferation was detected *in situ* based on Ki67 expression and analysed by confocal microscopy in 11 weeks old mice. Upper panels depict insulin and Ki67 staining in control littermates whereas the lower panels represent RIP-Cre *Dicer1^Δ/Δ^* pancreas. Although RIP-Cre *Dicer1^Δ/Δ^* islets have a clear decrease of insulin-producing cells compared to control littermates, no sign of increased proliferating Ki67 positive cells is observed.

To determine whether the observed phenotype of RIP-Cre *Dicer1^Δ/Δ^* was due to increased β-cell apoptosis that could be preceded or accompanied by an altered proliferative potential, we performed *in situ* TUNEL assay (Fig. 9A) and we stained pancreatic section with Ki67 specific antibodies (Fig. 9B). No detectable increased β-cell apoptosis could be observed in any of the strains at any time point. In addition, Ki67 staining did not differ in islets from RIP-Cre *Dicer1^Δ/Δ^* and control littermates (Fig. 9B).

## Discussion

In this work, we demonstrate for the first time that targeted disruption of the *Dicer1* gene specifically in β-cells leads to progressive reduction in insulin secretion, glucose tolerance and development of diabetes. Strikingly, 100% of RIP-Cre *Dicer1^Δ/Δ^* mice developed diabetes by 25 weeks of age indicating the importance of the miRNAs network for the proper function of insulin-producing cells and, in turn, for glucose metabolism. Both *in vitro* and *in vivo* studies demonstrate a decreased response to glucose stimulated insulin secretion in the RIP-Cre *Dicer1^Δ/Δ^* mice from 5 weeks of age. Prior to that, β-cell mass and function were not altered, suggesting that in our model disruption of *Dicer1*-dependent miRNA processing apparently does not affect the early stages of β-cell development. Indeed, insulin secretion in young mice, as well as the number of secretory granules, did not differ between RIP-Cre *Dicer1^Δ/Δ^* and littermates control mice.

While, our results partially affirm those observed by Morita et al. [31], who reports normal fetal and neonatal pancreatic development in *Dicer1*-hypomorphic mice that progress to morphological islet abnormalities only at 4 weeks of age. Our reported results contradict Lynn et al. [24] who observed no gross phenotype in mice driving *Dicer1* deletion using the RIP promoter. One explanation for the discrepancy with our study could be the different genetic background of the mouse strains [32] or the extent of *Dicer* deletion. However, neither *Dicer1* expression nor functional studies were performed in their mice with RIP-driven *Dicer1* deletion [24]. Pdx1-driven *Dicer1* deletion in β-cells had instead a profound effect in β-cell development [24]. The difference with our model is likely to be due to earlier expression of *Pdx1* [33] as compared with the *insulin* genes [32], [34], therefore earlier ablation of *Dicer1* may result in a more profound defect in β-cells reprogramming and differentiation.

What is the mechanism underlying β-cell mass reduction in the absence of the Dicer1 enzyme? Surprisingly, we could not observe any increase of β-cell apoptosis (measured by TUNEL *in situ*) or an altered β-cell proliferation based on Ki67 staining in RIP-Cre *Dicer1^Δ/Δ^* at any time point studied. However, we cannot exclude the possibility that β-cells undergo progressive apoptosis in a non-synchronized way posing a technical challenge in measuring cumulative cell death *in situ* at each specific time point. Alternatively, cell death could have occurred without the classical DNA single strands breaks measured by the TUNEL technique. Therefore, more studies are necessary to clarify this issue.

Interestingly, we observed not only reduction in the β-cell mass but a concomitant dramatic loss in insulin expression (Fig. 8) in the RIP-Cre *Dicer1^Δ/Δ^* mice with a reduced number of granules within β-cells (Fig. 7). This observation confirms the results recently published data where *Dicer1* was depleted in β-cells in adulthood [20]. That the regulation of insulin expression is controlled by miRNAs demonstrate the importance of these molecules in the control of insulin secretion. The reduced number of granules in the β-cell (Fig. 7B) might in part be due to the reduced insulin expression. However, the fact that no accumulation of empty granules was observed indicates that miRNAs also are necessary for the processing of insulin granules. The observed number of docked granules can be a cause of the reduced number of total granules. However, it cannot totally be excluded that depletion of *Dicer1* has a direct effect on the docking of granules due to that many miRNAs have been proven to be important for insulin exocytosis [35]. Indeed, the reduction in the number of docked granules (∼65%) in the RIP-Cre *Dicer1^Δ/Δ^* compared to control mice exceeds the reduction in the total number of granules (∼50%) indicating the importance of mature miRNAs in the regulation of the exocytotic process. Against this hypothesis is the observation that reduced glucose-stimulated insulin secretion in the RIP-Cre *Dicer1^Δ/Δ^* mice could entirely be explained by reduced islet insulin content (Fig. 2C) and glucose stimulated secretion as measured per insulin content was even significantly increased This would suggest that under the physiological condition of reduced insulin content, the RIP-Cre *Dicer1^Δ/Δ^* mice try to compensate by increasing processes involved in secretion. In such scenario, the reduced number of docked granules in the RIP-Cre *Dicer1^Δ/Δ^* mice is more likely associated with increased fusion of insulin granules (with less content in each granule) and failure of replenishment of docked granules.

In comparison with earlier published work where *Dicer1* deletion was driven by the Pdx-promotor [24] or in adulthood under the RIP-promotor [20] our data (where *Dicer1* is driven by the RIP-promotor earlier in life) demonstrate a broader phenotype. We observe not only reduction of insulin gene expression and concomitant reduced number of granules in the β-cells, but also altered islet morphology and reduced β-cells mass. This suggests that miRNAs control several important steps including insulin biosynthesis and β-cell survival. Further, *Dicer1*-dependent miRNAs are most likely controlling the islet architecture early in life although that the observed effects on β-cell survival are visible first around week 8 (See e.g. Fig. 4). Once *Dicer1* is deleted in adulthood islet architecture stays intact [20]. Although we were not able to demonstrate an increase in the frequency of apoptotic cells in the islets of RIP-Cre *Dicer1^Δ/Δ^* mice, we cannot rule out the possibility that β-cell loss is due to increased cell death. Our model is indeed characterized by a progressive loss of β-cell mass, which in time results in full blown diabetes. It is well known that apoptotic cells are very rapidly eliminated by scavenger cells and it is therefore likely that non synchronized apoptosis accompanied by a rapid phagocytosis and elimination of apoptotic cells have hampered the possibility of detecting the increase in beta cell death that should be expected based on the phenotype of these mice.

Finally, our results highlight the role of miRNAs in diabetes development. It is conceivable that the fine-tuning regulation of gene expression mediated by miRNAs might have important implication in diabetes pathogenesis also in humans [36]. In support, we have recently published data indicating a disturbed miRNA network in islets from human glucose intolerant donors [18] and in the GK-rat [22]. Our mouse model suggests that several miRNAs are involved in the development of diabetes. Overexpression of the β-cell specific miRNA, miR-375, in pancreatic islets reduce insulin secretion [17] and reduced β-cell mass was detected in the miR-375 knock-out mouse as well as hyperglycaemia [19]. Since our model is characterized by altered miRNAs processing, including miR-375, it is likely that the reduced expression of miR-375 in RIP-Cre *Dicer1^Δ/Δ^* mice (Fig. 8B) contributes to diabetes development although it appears clear that other miRNAs have to be involved in the pathophysiology of these mice. It remains unclear whether miR-375 knock-out mice develop overt diabetes. In contrast, 100% of the RIP-Cre *Dicer1^Δ/Δ^* mice developed diabetes. Thus, a number of miRNAs, including miR-375, contribute to efficient β-cell function and protection from diabetes. Defining the miRNA network used by β-cells to maintain glucose homeostasis will be, in the future, very useful to design new therapeutic strategies to prevent and eventually reverse diabetes development.

## Materials and Methods

### Ethical statement

All experiments were performed in compliance with relevant Swedish and institutional laws and guidelines and were approved by the local ethics committee in Malmö (Malmö-Lund animal research ethics committee; M133-10 and M78-08).

### Animals

Beta-cell specific *Dicer1* deficient mice (RIP-Cre *Dicer1^Δ/Δ^*) were generated by crossing RIP-Cre*^+/−^* mice [37] with *Dicer1* ^flox/flox^ mice, previously described [38], to obtain RIP-Cre*^+/−^ Dicer1^Δ^* ^/wt^ which were then crossed with *Dicer1* ^flox/flox^ mice. All mice used in the experiments carried the Cre transgene in heterozigosity and RIP-Cre*^+/−^ Dicer1^Δ^* ^/wt^ littermates were used as control mice (control). All mice were maintained on a 12-h light/dark cycle and fed *ad libitum* with standard chow and tap water at Malmö University Hospital animal facility.

### PCR

*Dicer1* ^flox/flox^ genotype was determined using the following PCR primers (5′-3′): AGTGTAGCCTTAGCCATTTGC and CTGGTGGCTTGAGGACAAGAC, the *wildtype* (wt) allele giving a 259 bp and the floxed allele a 309 bp PCR product. The Cre allele was identified by a 675 bp large PCR product amplified using the TGCCACGACCAAGTGACAGC and CCAGGTTACGGATATAGTTCATG primers. Deletion of the *Dicer1* exon 20–21 fragment was detected using the following PCR primers (5′-3′): AGTAATGTGAGCAATAGTCCCAG and CTGGTGGCTTGAGGACAAGAC, giving a product in both littermate controls and RIP-Cre *Dicer1^Δ/Δ^* mice of 309 bp. Complete deletion of *Dicer1* exon 20–21 results in the absence of the 259 bp and the floxed allele a 309 bp PCR product bands in RIP-Cre *Dicer1^Δ/Δ^* mice.

### Diabetes incidence

Mice were screened for blood glucose levels weekly starting from 3 weeks of age and were considered diabetic when glucose levels were ≥13.5 mmol/l on two consecutive measurements. The onset of diabetes was dated from the first of these sequential measurements. Tests were done at the same time daily to avoid variations due to feeding.

### *In vivo* glucose tolerance test

Control mice (n = 6) and RIP-Cre *Dicer1^Δ/Δ^* mice were subjected to *intraperitoneal* glucose tolerance test (ipGTT) at 4–5 weeks of age and at 13 weeks of age. Mice were anaesthetised by i.p. injections of Hypnorm (0.315 mg/ml Fetanyl, 10 mg/ml Fluanisone, VetaPharma) and Dormicum (5 mg/ml, Roche AB, Stockholm) diluted four times in sterile water; 50 µl of the Hypnorm/Dormicum mix was first injected 15 minutes before experiment subcutaneously and then repeated every 20 minutes i.p. The body warmth was maintained by red lamp. Mice were injected intraperitoneally (i.p.) with 0.9% NaCl solution containing 2 mg D-glucose / 1 g body weight. Blood samples for glucose measurements were taken at 0, 5, 10, 15, 30, 60 and 120 minutes after the glucose load.

### Islet isolation and *in vitro* insulin secretion assay

Mice were sacrificed by cervical dislocation and pancreatic islets were isolated by collagenase P digestion (Boehringer Mannheim, Bromma, Sweden). Insulin release *in vitro* was measured in static incubations [39]. Briefly, freshly isolated islets were preincubated for 30 minutes at 37°C in a Krebs-Ringer bicarbonate buffer supplemented with 1 mM glucose (pH 7.4). The medium was gassed with 95% O_2_ and 5% CO_2_ to obtain constant pH and oxygenation. Groups of 5 islets were then incubated in 0.2 ml Krebs-Ringer buffered solution supplemented with either 1 mM or 20 mM glucose for 60 minutes at 37°C. Immediately after incubation, an 80 µl aliquot of the medium was removed and the amount of secreted insulin was determined by ELISA (Rat Insulin ELISA-Kit, Mercodia, NC, USA). Insulin content was determined by RIA (radio immuno assay) after sonication and treatment with acid ethanol as previously described [39].

### Transmission Electron Microscopy

Pancreatic islets were harvested from 2 week and 11 week old β-cell-specific RIP-Cre *Dicer1^Δ/Δ^* mice and their control littermates and fixated in 2.5% glutaraldehyde for 1 h at 4° C. Thereafter they were treated with 1% osmium tetroxide, dehydrated and embedded in Durcupan (Sigma, Sweden) before being cut into 70–90 nm ultrathin sections. The sections were put on Cu-grids and contrasted with uranyl acetate and lead citrate before examined in a JEM 1230 electron microscope (JEOL-USA. Inc., MA, USA). Micrographs were analysed with respect to the intracellular granular distribution using similar methods that have been described elsewhere [40]. The apparent diameter of individual LDCVs was determined with the image processing software Scion Image (NIH freeware). The LDCV volume density, the docked LDCV surface density and LDCV profiles were calculated using in-house software programmed in MatLab (Matlab7).

### Histological and immunohistochemical analysis

After removal of the pancreata, one half of each pancreas was fixed with 4% formaldehyde and the other half rapidly frozen in *Tissue-Tek*® *O.C.T.™ Compound* (Sakura Finetek, Europe) on dry ice overnight, then stored at −80°C. Fixed tissues were processed for paraffin embedding. Serial 5 µm thick sections were prepared and stained. Paraffin-embedded sections were stained with hematoxylin/eosin (Sigma) to assess pancreatic islets histology and morphology in the experimental animals.

The immunofluorescence stainings were performed on paraffin sections or cryo sections. Paraffin sections were incubated with the following primary antibodies: guinea pig anti-insulin (1∶1000), guinea pig anti-glucagon (1∶600), rabbit anti-glucagon (1∶1000) (all from Eurodiagnostica, Malmö, Sweden) and rabbit anti-Ki67 (1∶200) (Novocastra Laboratories Ltd, Newcastle Upon Tyne, UK). Cryosections were fixed in acetone for 7 min, blocked with BSA and incubated with primary antibodies (or mixtures, if double-stained) at 4°C overnight, washed three times for 5 min in PBS, then incubated with FITC-conjugated anti guinea pig IgG (1∶200) and Cy3-conjugated anti-rabbit IgG (1∶300) (both from Jackson Immunoresearch Laboratories Inc, West Grove, PA) secondary antibodies for 45 minutes at room temperature. Cell nuclei were visualized after mounting with ProLong Gold antifade reagent with DAPI (Invitrogen, Carlsbad, CA, USA). Immunostainings were visualized using a Zeiss LSM510 Confocal microscope and laser excitations wavelengths of 488 nm and 543 nm. To control the specificities of the immunostaining procedures, the primary antibodies were replaced by normal serum from the appropriate species or by PBS. Quantification of insulin- and glucagon positive cells was measured using Zeiss LSM 510 program and was expressed as the ratio between insulin – and glucagon fluorescence in each islet.

### Optical Projection Tomography (OPT)

The OPT technology was used to visualize the total ß-cells in the experimental pancreas. In brief, pancreas from RIP-Cre *Dicer1^Δ/Δ^* and control mice were dissected, cleaned of residual fat tissue and immediately submersed into freshly made 4% paraformaldehyde (Sigma P6148) in PBS for 3 h at 4°C, washed in PBS stepwise transferred to 100% methanol (MeOH) and stored at −20°C. Subsequently the pancreatic samples were stained with primary antibody Guinea Pig anti-Insulin (A0564, Dako) and secondary antibody goat anti GP Alexa Fluor 594 (A-11076, Invitrogen). OPT scannings of the pancreatic samples were carried out using the Bioptonics 3001 OPT M scanner (Bioptonics), with exciter D560/40× and emitter E610lpv2 filter (Chroma) as described previously [26]. Tomographic re-constructions were generated by using the NRecon V1.6.1.0 (Skyscan, Belgium) software and reconstructed images were further assessed using Bioptonics viewer V2.0 (Bioptonics) as previously described [26]. Further quantification for islet number was done using ImageJ 1.45n software. Briefly, image stack was opened using import/image sequence and saved as .avi file. After setting the scale globally the image channels were splitted and only the channel having the insulin staining was further used for quantitative analysis. This was first converted to 8 bit and then threshold was set to cover all the islets throughout the stack. 3D object counter plugin was used to count the number of islets. Possible artifacts were ruled out manually by using the synchronize tool and going through each slide to get the correct number of particles. The quantification of the beta cell volume was done by Volocity v5.2.1 (PerkinElmer) as per published method [26], [27]. Possible artifacts were omitted manually by going through slides to generate the correct beta cell volumes.

### β-cell sorting

β-cell sorting was performed based on Newport Green-Ac staining as described by B. Lukowiak et al. with some modifications [41]. Briefly, islets from control littermates and RIP-Cre *Dicer1^Δ/Δ^* mice were incubated in a calcium-free buffer in 37°C for 12 minutes and then dissociated into single cell suspension by repeated pipetting. Islet cells were then incubated for 90 minutes in Opti-MEM minimal medium (Gibco, Invitrogen, Carlsbad, CA, USA) supplemented with 2.8 mM D-glucose and 1% L-glutamin. Subsequently, the cells were incubated with 1 µM Newport Green Ac-ester (Invitrogen, Carlsbad, CA, USA) and 1 µl/ml Pluronic F-127 dispersing agent (Invitrogen, Carlsbad, CA, USA) in 37°C for 30 minutes, to facilitate Ac-ester cell loading. Finally, cells were washed in indicator-free Opti-MEM medium and incubated for a further 30 minutes in 37°C to allow complete de-esterification of intra-cellular AM-esters. Newport Green-Ac stained islets cells were then subjected to cell sorting using a standard equipped fluorescence activated cell sorting (FACS) instrument (FACSaria, BD Biosciences, San Diego, CA, USA). Dead-cell discrimination was performed by staining cells with 7AAD (BD Pharmingen, USA). Cells were kept on ice during sorting procedures. Excitation was performed with the blue laser at 488 nm, and the high Newport Green positive cells were sorted in the Fl.1 channel. A fraction of sorted cells was placed on glass slides to confirm purity by immunofluorescence microscopy and viability while the rest of the cells were used for RNA purification as described below. Cell sorting analysis was performed using the Diva 6.1 software (BD Biosciences, San Diego, CA, USA).

### Immunofluorescence microscopy of sorted β-cells

Sorted β-cells were cultured for 48 hours on cover glasses in RPMI 1640 (Gibco, Invitrogen) with Peni-Strep, 10% FCS and 1% L-glutamin to attach on the glass for later immunohistochemistry staining. Cell fractions were carefully washed with PBS after culture, and fixed with 4% paraformaldehyde for 20 minutes at room temperature. The cells were then permeabilized and blocked with PBS containing 0.1% Triton X-100 and 10% BSA for 45 minutes at room temperature. After blocking, the cells were incubated with 10 µg/ml monoclonal rat anti-mouse insulin IgG antibody (R&D Systems) in 4°C over night, followed by goat anti-rat IgG-Phycoerythrin secondary antibody / or goat anti-rat Alexa-456. Mounting was performed with ProLong Gold antifade reagent with DAPI (Invitrogen, Carlsbad, CA, USA).

### Flow cytometry analysis of islet cells

Mouse islets were dissociated into cell suspension and fixed with Fixation/Permeabilization Buffer for 1.5 h, 4°C. After washing twice with Permeabilization/Wash Buffer (eBiosciences, San Diego, CA, USA), islet cells were stained with monoclonal rat-anti mouse insulin-phycoerythrin (PE) and/or monoclonal rat-anti mouse glucagon-allophycocyanin (APC) antibody (both from R&D Systems, MN, USA). Antibodies were direct-conjugated with Lightning Link-PE or APC (InnovaBiosciences, Cambridge, UK) for flow cytometry purposes before use. Islet cells were incubated in the dark with antibodies for 30 minutes at 4°C and then washed twice again with Permeabilization/Wash Buffer before resuspend them in staining buffer and acquired by flow cytometry on FACSCalibur Instrument and data were analyzed using CellQuest software (both from Becton Dickinson, San Jose, CA, USA).

### Quantitative PCR (qPCR)

Total RNA from whole brain and FACS-sorted control and RIP-Cre Dicer1*^Δ/Δ^*-β-cells was extracted using the Qiagen miRNeasy isolation kit according to manufacturer's protocol (Qiagen, Hilden, Germany). The obtained RNA was quantified by NanoDrop ND-1000 (Thermo Fisher Scientific, USA) and subjected to reverse transcription using Quantitect Reverse Transcription Kit (Qiagen, Hilden, Germany), according to the manufacturer's suggested reaction conditions. qPCR was performed in triplicates using Taqman assays (Applied Biosystems, Foster City, CA, USA) for mouse insulin, mouse Dicer exon boundary 20–21, and mouse pri-miR-375. Mouse Hprt1 and cyclophilin were used as endogenous controls. For mature miR-375, stem-loop qPCR was performed normalized against snoRNA-202 and snoRNA-412. All runs were performed on ABI 7900 HT Fast Real-Time PCR Detection System (Applied Biosystems, Foster City, CA, USA).

### Global microRNA expression profiling

Total RNA from sorted β-cells of wildtype, *Dicer1^flox/flox^* and RIP-Cre *Dicer1^Δ/Δ^* mice was extracted using the Qiagen miRNeasy isolation kit (Qiagen, Hilden, Germany). 50 ng of total RNA from each sample were labeled with the miRCURY™ Hy3™ dye using the accompanying kit and hybridized on the miRCURY™ LNA Array (v.11.0) according to manufacturer's protocol for Maui hybridisation chamber (Exiqon, Vedbaek, Denmark). Images were acquired using the Axon® GenePix 4000B scanner and GenePix software. After background subtraction from foreground signals, intensities within arrays were normalized using the loess normalization algorithm while quantile normalization was used between arrays as implemented in the web-fronted R-based array analysis tool, CARMAWeb 1.4. [42]. Only signals >50 were considered for analysis. The log_2_ values were used to generate heatmap using TreeView (http://jtreeview.sourceforge.net).

### *In situ* Apoptosis detection

To identify apoptotic cells within the pancreatic islets, the Terminal Deoxynucleotidyl Transferase (TdT)-mediated dUTP end labelling (TUNEL) assay was used in paraffin embedded sections. The assay was performed accordingly to the manufacturer recommendation (TACS TdT-Fluorescein In Situ Apoptosis Detection Kit, R&D System Europe, Ltd, Abingdon, UK).” Nuclease-treated pancreas section from littermate control mice was used as positive control for TUNEL staining.

### Statistical analysis

Differences in the incidence of diabetes were assessed using Kaplan–Meier analysis. Statistical significances of differences between means were estimated by Student's *t*-test. Values of P<0.05 were considered significant.

## Supporting Information

Figure S1 **β-cell specific knock-out of** ***Dicer1*** **does not affect Dicer1 expression in brain or feeding behaviour.** **A.** Dicer1 expression in brain from RIP-Cre *Dicer1^Δ/Δ^* mice and control littermates (control). Expression is normalized to HPRT and cyclophilin expression levels. **B.** Weight of 6 week-old RIP-Cre *Dicer1^Δ/Δ^* mice and control littermates (Control) measured during 6 days. **C.** Food consumption during a 5 days period in 6 week old RIP-Cre *Dicer1^Δ/Δ^* mice and control littermates (Control).
(PDF)

Figure S2 **Electron micrograph of β-cells from 2 week old control (top) and RIP-Cre** ***Dicer1^Δ/Δ^*** **(bottom) mice.** In the *left* images (low magnification), the plasma membrane surrounding the analyzed β-cell is marked by a black line. The area within the dotted rectangle is high-lighted in images (higher magnification) to the *right*. Docked granules are marked with black arrows in the images to the right. Scale bars 2 µm (*left images*) and 0.5 µm (*right images*). β: β-cell; α: α-cell; N: nucleus; g: granule; m:mitochondria.
(PDF)

Figure S3 **Global miRNA profiling in sorted β-cells.** Pancreatic islets were isolated from 7 RIP-Cre *Dicer1^Δ/Δ^* and 7 littermate control mice. β-cells were sorted using flow cytometry to >98% purity. Total RNA was extracted and used for PCR and array hybridization. This image shows the results from the array. A combined 60 miRNAs exhibited detectable signals from the arrays. 26 were not detected in the knockout including the most abundant islet miRNAs, miR-375 and miR-7a. (black to yellow: normalized array signal; grey: no signal).
(PDF)
