# Supplementary figures and images for: Beta-Cell Specific Deletion of Dicer1 Leads to Defective Insulin Secretion and Diabetes Mellitus
Source: PLoS One. 2011 Dec 27;6(12):e29166. doi: 10.1371/journal.pone.0029166 (PMC3246465; doi:10.1371/journal.pone.0029166)

**Figure S1**

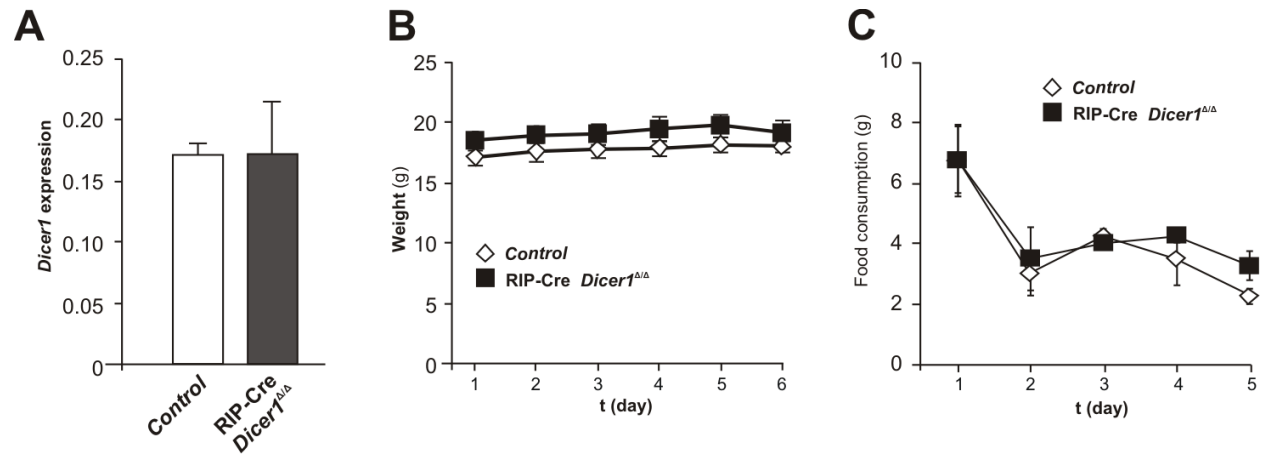

Supplement: Figure S1 — β-cell specific knock-out of Dicer1 does not affect Dicer1 expression in brain or feeding behaviour. A. Dicer1 expression in brain from RIP-Cre Dicer1Δ/Δ mice and control littermates (control). Expression is normalized to HPRT and cyclophilin expression levels. B. Weight of 6 week-old RIP-Cre Dicer1Δ/Δ mice and control littermates (Control) measured during 6 days. C. Food consumption during a 5 days period in 6 week old RIP-Cre Dicer1Δ/Δ mice and control littermates (Control). (PDF) [file pone.0029166.s001.pdf]

**Figure S2**

***Control***

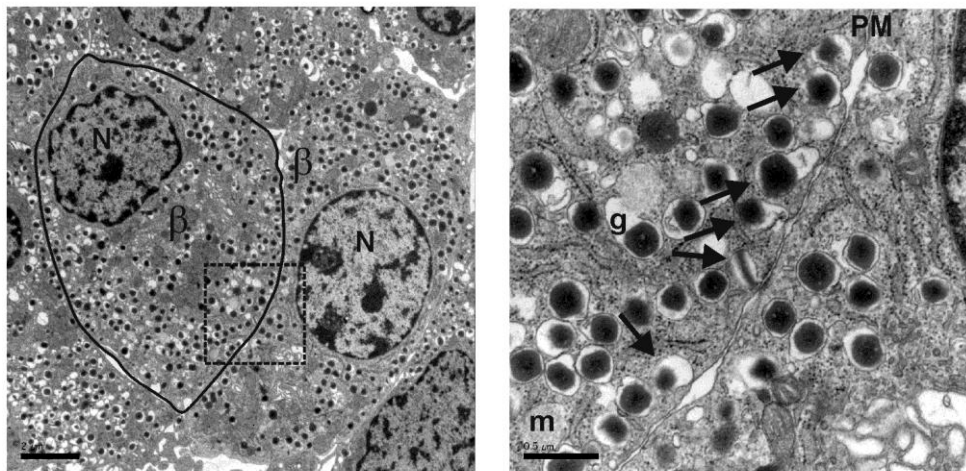

***RIP-Cre  $Dicer1^{\Delta/\Delta}$***

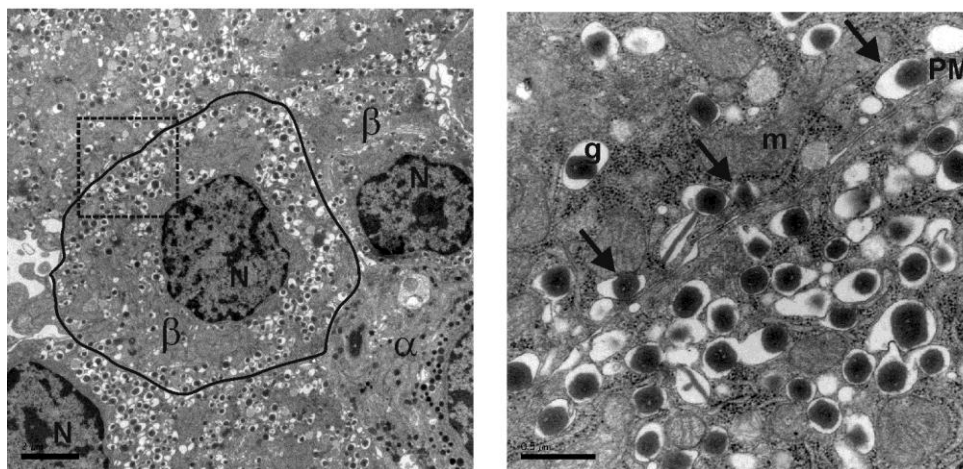

Supplement: Figure S2 — Electron micrograph of β-cells from 2 week old control (top) and RIP-Cre Dicer1Δ/Δ (bottom) mice. In the left images (low magnification), the plasma membrane surrounding the analyzed β-cell is marked by a black line. The area within the dotted rectangle is high-lighted in images (higher magnification) to the right. Docked granules are marked with black arrows in the images to the right. Scale bars 2 µm (left images) and 0.5 µm (right images). β: β-cell; α: α-cell; N: nucleus; g: granule; m:mitochondria. (PDF) [file pone.0029166.s002.pdf]

Figure S3

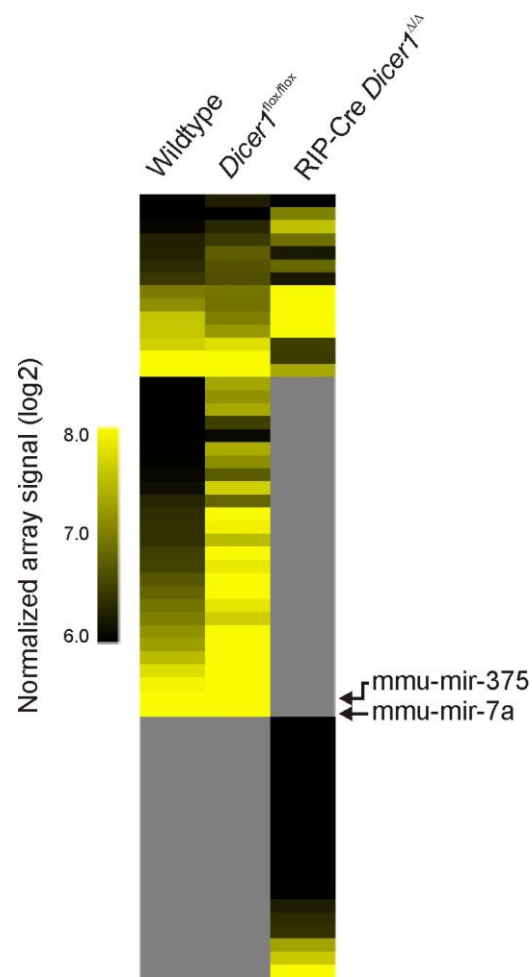

Supplement: Figure S3 — Global miRNA profiling in sorted β-cells. Pancreatic islets were isolated from 7 RIP-Cre Dicer1Δ/Δ and 7 littermate control mice. β-cells were sorted using flow cytometry to >98% purity. Total RNA was extracted and used for PCR and array hybridization. This image shows the results from the array. A combined 60 miRNAs exhibited detectable signals from the arrays. 26 were not detected in the knockout including the most abundant islet miRNAs, miR-375 and miR-7a. (black to yellow: normalized array signal; grey: no signal). (PDF) [file pone.0029166.s003.pdf]
